# Saudi Female Sexual Dysfunction After Bariatric Surgery: A Cross-Sectional Survey

**DOI:** 10.7759/cureus.53196

**Published:** 2024-01-29

**Authors:** Abdulhamid Alharbi, Taif H Alomar, Taif S Alharbi, Ranad S Alamri, Abdulaziz K Alharbi, Braa S Almadani

**Affiliations:** 1 General Surgery Obesity Center, King Fahad General Hospital, Medina, SAU; 2 Medicine, College of Medicine, Taibah University, Medina, SAU; 3 Medicine and Surgery, College of Medicine, Taibah University, Medina, SAU; 4 Obstetrics and Gynecology, Ohud Hospital, Medina, SAU

**Keywords:** female sexual function index, obesity, saudi arabia, bariatric surgery, female sexual dysfunction

## Abstract

Background

Obesity disrupts the equilibrium of sexual hormones, resulting in decreased sexual desire, arousal, and orgasm. The aim of this study was to investigate the effects of substantial weight loss after bariatric surgery on sexual function, psychological health, and the overall quality of sexual life in a group of Saudi females.

Method

The study used a cross-sectional design and was conducted at King Fahad Hospital in Medina, Saudi Arabia. The study included adult female patients who had previously undergone bariatric surgery. We used the Sexual Quality of Life for Female (SQoL-F) and the Female Sexual Function Index (FSFI) questionnaires to collect data. The study was extended from January 1, 2021, to December 30, 2022.

Results

A total of 100 participants were included in this study, all the samples underwent vertical sleeve gastrectomy, their mean age was 36.7±9.3, 94% (n=94) of the respondents had high school education or above, 50.0% (n=50) were unemployed, and around 13% (n=13) of the samples had a psychiatric history. Surgery complications were reported in 10% (n=10), which were reported as esophagitis (n=4), gastric ulcer (n=2), gastric stricture (n=1), infection (n=2), and leakage (n=1). The median of the FSFI and SQoL-F was 47.0 and 24.5, respectively. Approximately 66% of the respondents agreed that their sexual lives improved after surgery, 22% did not feel any difference before and after surgery, and 9% witnessed deterioration. In total, 61.0% had female sexual dysfunction (FSD) (25% had no dysfunction afterward, 45% had mild dysfunction, 27% suffered mild to moderate dysfunction, and only 2% had severe dysfunction). Regarding SQoL-F, the mean score was 5.59 for sexual repression, 6.1 for self-worthlessness, 18.56 for sexual and relationship satisfaction, and 16.4 for psychological feelings.

Conclusions

Bariatric surgery was associated with the improvement of female sexual function.

## Introduction

Female sexual dysfunction (FSD) is a complex issue marked by changes in the cycle of sexual responses and discomfort during or after a relationship [1]. Initially perceived as primarily psychological, it is now understood as a multifaceted problem involving neurobiological, hormonal, and psychosocial factors that affect females' well-being, quality of life, and relationships [2]. Up to 43% of females experience some form of FSD [3], with higher rates among older individuals and those with lower estrogen levels [4]. Medical conditions (cancer, kidney failure, multiple sclerosis, heart disease, bladder problems, and obesity) increase the risk of FSD [5].

Historically, sexual dysfunction was primarily associated with males, but today, it is acknowledged as a significant health concern for females as well [5]. FSD encompasses issues related to desire, arousal, orgasm, pain, lubrication, and satisfaction in females [6]. The prevalence of FSD in females living in Islamic countries varies widely; a study in Iran found that FSD ranges from 25% to 63% [7]. In a clinic-based survey in Riyadh, 200 Saudi females aged 18-50 years were assessed for female sexual function using the Arabic version of the Female Sexual Function Index (ArFSFI). Most of the participants were satisfied with their spouse's sexual ability, but 60% were at risk for FSD. Those with FSD had lower scores in arousal, desire, and orgasm domains [8]. Rouzi et al. [9] assessed the prevalence of sexual dysfunction in female healthcare providers in Jeddah, Saudi Arabia, comprising both Saudi and non-Saudi participants. While desire and arousal scores showed no significant differences, non-Saudi females scored significantly higher in lubrication, orgasm, satisfaction, and pain domains. Non-Saudi females also achieved a significantly higher overall score.

Obesity disrupts the equilibrium of sexual hormones, resulting in decreased sexual desire, arousal, and orgasm [10]. Furthermore, it increases the risk that people develop cardiometabolic conditions such as type 2 diabetes, dyslipidemia, and hypertension (HTN), which have established associations with reduced sexual activity [11]. Additionally, people with obesity are more susceptible to experiencing problems related to anxiety and depression, which are believed to have a direct or indirect association with sexual function [12]. This underscores the multifaceted impact of obesity on sexual health and overall well-being [13]. Notably, obese females exhibit a higher prevalence of FSD, ranging from 50% to 86%, and this is correlated with a higher body mass index (BMI), which significantly impacts their sexual quality of life [14]. Despite the importance of promoting sexual function in obese females with sexual dysfunction, which is a crucial aspect of females' healthcare [15], there is limited evidence regarding the specific impact of weight loss on improving sexual function, particularly within the Arab world.

We hypothesize that significant weight loss after bariatric surgery will lead to improvements in sexual function, psychological well-being, and the overall quality of sexual life in obese Saudi females experiencing sexual dysfunction. The purpose of this study was to investigate the effects of weight loss after bariatric surgery on sexual function and the overall quality of sexual life in a group of Saudi females.

## Materials and methods

Study design

The study used a cross-sectional design and was conducted at King Fahad Hospital in Medina, Saudi Arabia.

Study participants

The study focused on adult married female patients who had previously undergone bariatric surgery. The inclusion period for the study participants was extended from January 1, 2021, to December 30, 2022. The participants were recruited through a systematic random sampling method. A monthly list of patients undergoing bariatric surgery was compiled, and a sampling frame was created by dividing the total number of cases by the desired sample size. A sampling interval was established, and the initial case was chosen randomly. Subsequent cases were selected by adding the sampling interval to the last case chosen.

Data collection

Data collection was facilitated through the completion of a structured questionnaire, which was divided into three distinct sections.

Section 1: Sociodemographic Data

This section collected sociodemographic information, including the participants' age, marital status, and occupational status; the presence of comorbidities; and their body weight.

Section 2: Surgery-Related Information

The second section of the questionnaire collected detailed data about surgical procedures. It included information such as the type of surgery each participant had undergone, the date of the surgery, and any complications that arose in the postoperative period.

Section 3: The Evaluation of Female Sexual Function

The third section involved the administration of two standardized assessment tools.

The Female Sexual Function Index (FSFI): It is a widely used tool to assess various aspects of female sexual function. It covers sexual desire, arousal, lubrication, orgasm, satisfaction, and pain through a 19-item questionnaire. Responses are rated on a five-point scale, with lower scores indicating lower sexual functioning. The FSFI is structured into six domains, each consisting of a different set of questions. These domains are desire (two questions), arousal (four questions), lubrication (four questions), orgasm (three questions), satisfaction (three questions), and pain (three questions). This index is used to assess various aspects of female sexual functioning [16]. A total score of 26.55 or less in FSFI, with a maximum score of 36, is indicative of female sexual dysfunction (FSD) [17]. This score serves as a threshold for identifying sexual dysfunction in females. It was developed by Rosen et al. in 2000 using both the clinical and control groups, aiming to provide a validated self-report instrument for researching female sexuality. The FSFI exhibits high reliability with strong internal consistency and test-retest reliability, as confirmed by various studies [18].

The Sexual Quality of Life for Female (SQoL-F) questionnaire: It is a concise tool designed to evaluate the association between female sexual dysfunction and overall quality of life. It was developed by Symonds et al. in 2005. The questionnaire draws inspiration from Spitzer's Quality of Life model, which encompasses physical, emotional, psychological, and social components [19]. The SQoL-F comprises 18 items, and the respondents provide their feedback using a six-point scale, ranging from "completely agree" to "completely disagree." Each item can be scored within the range of 1-6 or 0-5, resulting in total scores ranging from 18 to 108 or 0 to 90. In this scoring system, higher scores indicate a higher level of female sexual quality of life, reflecting improved sexual well-being and emotional satisfaction [19]. The SQoL-F questionnaire is structured into four distinct factors, each serving to assess different aspects of female sexual function and its impact on the quality of life. Factor 1, termed "psychosexual feelings," focuses on elements related to emotional and psychological dimensions, involving seven specific items. Factor 2, "sexual and relationship satisfaction," evaluates satisfaction levels in sexual and relational aspects through five items. Factor 3, labeled "self-worthlessness," measures self-esteem and feelings of self-worth, encapsulated within three items. Factor 4, "sexual repression," examines issues related to sexual inhibition, comprising three items. This multifactorial structure enables a comprehensive assessment of the complex interplay between female sexual function and overall quality of life [20].

Bias considerations

Through the use of random sampling techniques, selection bias was attempted to be minimized. The participants were urged to provide accurate and sincere answers during data collection to reduce information bias. To increase the dependability of responses, privacy and confidentiality were maintained.

Outcomes

The primary outcome of this study was the impact of bariatric surgery on the sexual functioning of postoperative female patients. The secondary outcomes included the prevalence of comorbidities, surgical complications, and the sociodemographic factors that may influence postoperative sexual function.

Data processing and statistical analysis

The data collected were carefully examined, coded, and entered into a safe electronic database. To guarantee the quality and consistency of the data, skilled workers entered and processed the data. Procedures for anonymization were used to protect the participants' privacy and identity. Descriptive statistics were used to summarize sociodemographic characteristics and surgical details. The scores of the FSFI and SQoL-F questionnaires were analyzed using t-tests or chi-square tests. Significance was set at a p-value of less than 0.05 for statistical tests. Statistical analyses were performed using the R software (R Foundation for Statistical Computing, Vienna, Austria).

Ethical considerations

The study received ethical approval from the Institutional Review Board (IRB) of the General Directorate of Health Affairs in Medina, using the reference number 23-072. To preserve the participants' confidentiality, strict measures were adopted. The confidentiality of each participant's identity and personal data was maintained. The study was carried out in compliance with accepted ethical norms and recommendations. To maintain anonymity, all participant information, including name and contact information, was deidentified. The dataset was only accessible to authorized members of the research team, and all information was securely kept to prevent illegal access. A formal informed consent form was signed by each participant.

## Results

A total of 100 participants were included in this study. All samples were subjected to vertical sleeve gastrectomy. The mean age of the study participants was 36.7±9.3 years. Of the studied participants, 94.0% of the respondents had secondary school education or higher, and only 13% (n=13) were below the secondary level. Half of the sample (50.0%, n=50) were unemployed, 45% (n=45) were employed, and the rest were retired (4%, n=4). Around 13% of the sample had a psychiatric history. Moreover, 24% of the respondents had medical histories such as asthma, diabetes mellitus (DM), hypertension (HTN), and dyslipidemia. The complications of surgery were reported in 10% (n=10), which were reported as esophagitis (n=4), gastric ulcer (n=2), stricture gastric (n=1), infection (n=2), and leakage (n=1) (Table 1).

**Table 1:** Sociodemographic characteristics of the studied participants (n=100) OCD: obsessive-compulsive disorder

Characteristics		N (%)
Age category	19-28	19 (19%)
	29-38	41 (41%)
	39-48	24 (24%)
	49+	16 (16%)
Level of education	Illiterate	1 (1%)
	Elementary school	5 (5%)
	Secondary school	33 (33%)
	Bachelor's	54 (54%)
	Master's	7 (7%)
Occupation	Employee	45 (45%)
	Retired	4 (4%)
	Unemployed	50 (50%)
Do you have any medical history	Yes	23 (23%)
	None	77 (77%)
Do you have any psychiatric history	Anxiety	3 (3%)
	Depression	6 (6%)
	OCD	2 (2%)
	Somatic symptom disorder	1 (1%)
	None	88 (88%)
Complication after surgery	No	90 (90%)
	Yes	10 (10%)

**Figure 1:**
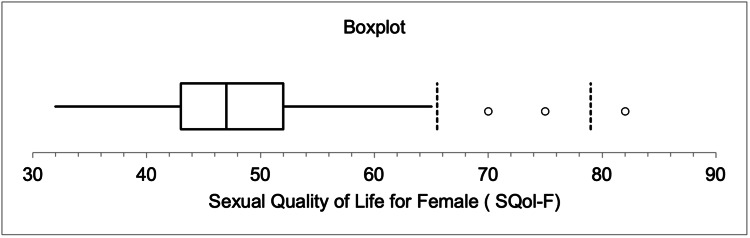
Boxplot of summary statistics related to Sexual Quality of Life for Female (SQoL-F)

**Figure 2:**
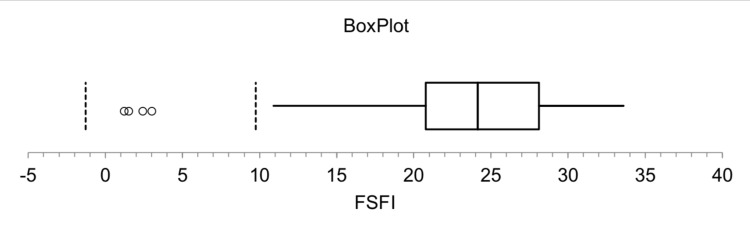
Boxplot of summary statistics related to the Female Sexual Function Index (FSFI) domain The data has been represented as median (IQR) IQR: interquartile range

Figure 1 and Figure 2 summarize the summary statistics of the two calculated scores (SQoL-F and FSFI) for the respondents. Boxplots revealed that the median of SQoL-F was 47, with three outliers above the maximum point of 82. The median of FSFI was 24.5 (in the mild disfunction category), meaning that 50% of the respondents had mild or no disfunction with three outlier observations less than or close to the minimum point (1.2).

**Figure 3:**
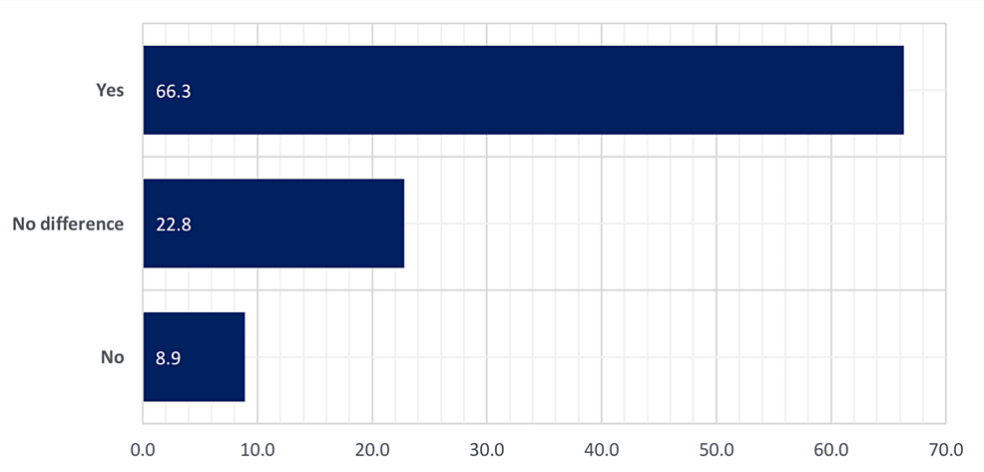
Relative distribution of females according to their opinion about the enhancement of sexual life after surgery The data has been represented as N (%)

Figure 3 presents the percentage distribution of the respondents. Around 66% of the respondents agreed that their sexual lives improved after surgery, 22% did not feel any difference before and after surgery, and 9% did not see any improvement.

**Figure 4:**
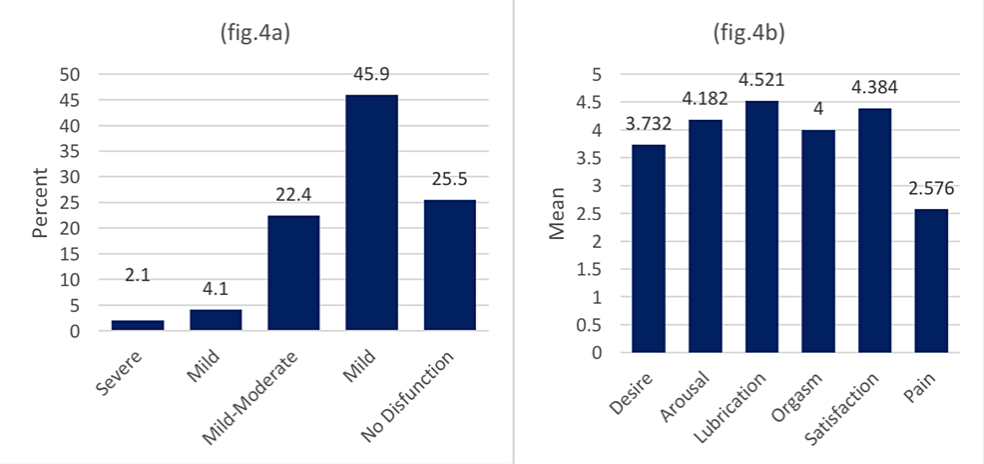
Relative distribution of females according to the disfunction categories of FSFI (a) and the mean score for each domain (b) The data has been represented as N (%) (a) and mean±SD (b) FSFI: Female Sexual Function Index

On the other hand, Figure 4 shows the categories of FSFI after disaggregating the score according to the literature. Based on the cutoff point at 26.55, 61.0% had FSD. Twenty-five percent of the respondents had no dysfunction after the surgery, 45% had mild dysfunction, 27% suffered from mild to moderate dysfunction, and only 2% had severe dysfunction. The mean score of FSFI was 2.6 for pain, 3.7 for desire, 4 for orgasm, 4.2 for arousal, and 4.3 for satisfaction. The highest was 4.5 for lubrication.

**Figure 5:**
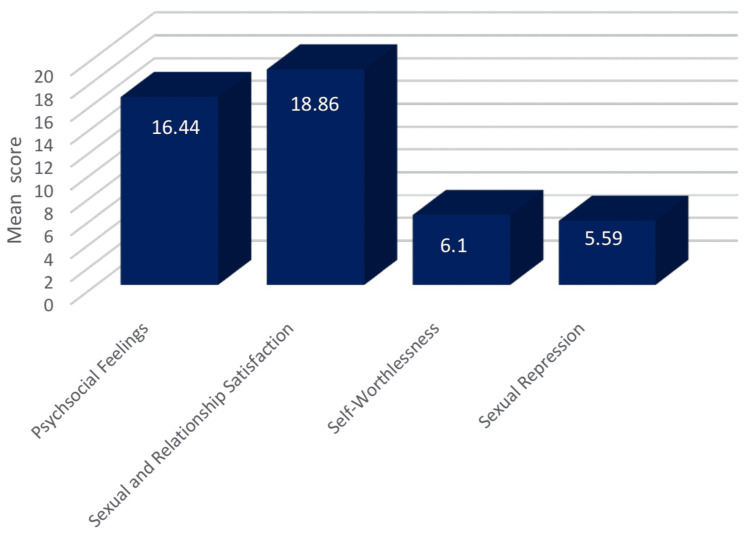
The mean of each domain in Sexual Quality of Life for Female The data has been represented as mean±SD

The mean score for each domain of SQoL-F is shown in Figure 5; it is as follows: 5.59 for sexual repression, 6.1 for self-worthlessness, 18.56 for sexual and relationship satisfaction, and 16.4 for psychological feelings (Figure 5).

Table 2 presents data on various factors and their relationship with perceived sexual function. In "complication after surgery," there was a significant difference between the responders (p=0.001), suggesting that complications are associated with perceived self-improvement after surgery. Other factors were not statistically significant.

**Table 2:** The distribution of the respondents according to their characteristics and sexual dysfunction severity The data has been represented as N (%); p<0.05 is considered significant

Label	Levels	Deteriorated	No difference	Improved	P-value
Age before the gastric sleeve (binned)	19-28	1 (5.3)	4 (21.1)	14 (73.7)	0.306
	29-38	2 (4.9)	8 (19.5)	31 (75.6)	
	39-48	2 (8.3)	8 (33.3)	14 (58.3)	
	49+	4 (25.0)	3 (18.8)	9 (56.2)	
Complication after surgery	No	4 (4.4)	22 (24.4)	64 (71.1)	0.001
	Yes	5 (50.0)	1 (10.0)	4 (40.0)	
Education after the gastric sleeve	Bachelor's degree or higher	5 (8.1)	15 (24.2)	42 (67.7)	0.246
	High school or less	4 (10.5)	8 (21)	26 (68.5)	
Social status after the gastric sleeve	Employee	4 (8.9)	12 (26.7)	29 (64.4)	0.920
	Unemployed	5 (9.8)	10 (19.6)	36 (70.6)	
	Retired		1 (25.0)	3 (75.0)	
Age before the gastric sleeve	19-28	1 (5.3)	4 (21.1)	14 (73.7)	0.306
	29-38	2 (4.9)	8 (19.5)	31 (75.6)	
	39-48	2 (8.3)	8 (33.3)	14 (58.3)	
	49+	4 (25.0)	3 (18.8)	9 (56.2)	
Age after the gastric sleeve	21-30	1 (5.0)	4 (20.0)	15 (75.0)	0.559
	31-40	2 (4.5)	11 (25.0)	31 (70.5)	
	41-50	4 (17.4)	6 (26.1)	13 (56.5)	
	51+	2 (15.4)	2 (15.4)	9 (69.2)	
Chronic diseases	No	6 (7.5)	18 (22.5)	56 (70.0)	0.478
	Yes	3 (15.0)	5 (25.0)	12 (60.0)	
Psychiatric history	No	8 (8.9)	20 (22.2)	62 (68.9)	0.764
	Yes	1 (10.0)	3 (30.0)	6 (60.0)	

## Discussion

Sex is a fundamental aspect of human existence and plays a crucial role in an individual's reproductive life [21]. This function involves the integration of physical, emotional, and psychological factors, and it significantly impacts an individual's overall quality of life [22]. Female sexual functioning encompasses the ability to achieve sexual arousal, lubrication, orgasm, and overall satisfaction during sexual experiences [23]. The study aimed to investigate the impact of substantial weight loss after bariatric surgery on sexual function, psychological health, and the overall quality of sexual life in a specific group of Saudi females.

The main study findings

In the study, a sample of females underwent vertical sleeve gastrectomy, and 94% had higher education. Most were unemployed, with a small percentage having medical histories. The complications of surgery were reported in 10% of the participants, which included esophagitis, gastric ulcers, and infections. The median SQoL-F score was 47, and the median FSFI score was 24.5. Most of the respondents felt that their sexual lives improved after surgery, and 66% stated this. The mean score for FSFI ranged from 2.6 for pain to 4.5 for lubrication. Complications were associated with a significant difference between the "no" and "yes" groups, suggesting a relationship between these factors.

Role of bariatric surgery in improving sexual dysfunction

FSD is a prevalent health issue that has not received adequate attention and research. Additionally, many females feel uncomfortable discussing matters related to their sexuality and sexual health with healthcare professionals. This challenge is more pronounced in Eastern countries, where such topics are often considered taboo. There is also a notable scarcity of published reports on the risk of FSD in Saudi Arabia.

A study at The Miriam Hospital in Providence, Rhode Island, examined if FSD resolves after bariatric surgery. Fifty-four females underwent two types of bariatric surgery: laparoscopic adjustable gastric banding (LAGB) and Roux-en-Y gastric bypass (RYGB). The results showed that 63% of females had scores indicative of FSD before surgery, but 68% had resolved six months later. Factors such as marriage, younger age, and worse preoperative sexual function were associated with greater improvements. Bond et al. [24] investigated the impact of weight loss on improving sexual function in Pakistan. In comparison to the current study, 300 married females were surveyed using the FSFI. The results showed significant improvements in FSFI scores for the participants with weight loss between 2% and 5% of their initial body weight and for those with weight loss of more than 5%. The study highlights the need for further research on the impact of weight loss on sexual function in developing countries [25]. In this study, we found that there is a high prevalence of FSD, affecting approximately 61.0% of the participants. Pain and desire were the most affected domains of sexual function. A similar study conducted in Riyad, Saudi Arabia, addressed a prevalence of 60%. These results are consistent with similar studies conducted in Egypt [26] and Japan [27]. However, we observed slightly lower rates of FSD in comparison to studies conducted in Turkey [28], Iran [7], and the United States [29]. These variations in the prevalence of FSD in different countries can be attributed to a variety of factors, including medical and psychological aspects; the influence of socioeconomic, cultural, and racial differences; the specific clinical definitions used for each type of dysfunction; and the characteristics of the samples examined.

Strengths and limitations

This study presents several strengths, including the use of standardized assessment tools, a structured questionnaire, a relatively large sample size of females who underwent bariatric surgery, and an ethical emphasis on privacy and confidentiality during data collection. However, it is limited by its cross-sectional design, which restricts the ability to establish causation; potential response bias; self-reported data that may be influenced by recall bias; its single-center nature; a relatively non-follow-up nor baseline assessment; and the absence of a control group. These limitations should be considered when interpreting the findings, and they highlight areas for future research to provide a more comprehensive understanding of the impact of bariatric surgery on postoperative sexual functioning.

## Conclusions

In the study, a sample of females underwent vertical sleeve gastrectomy, and 94% had higher education. Most were unemployed, with a small percentage having medical histories. Surgery complications were reported in 10% of the participants, which included esophagitis, gastric ulcers, and infections. The median SQoL-F score was 47, and the median FSFI score was 24.5. Most of the respondents felt that their sexual lives improved after surgery, and 66% stated this. The mean score for FSFI ranged from 2.6 for pain to 4.5 for lubrication. Complications were associated with a significant difference between the "no" and "yes" groups, suggesting a relationship between these factors.
